# Mental Health of Tourism Employees Post COVID-19 Pandemic: A Test of Antecedents and Moderators

**DOI:** 10.3390/ejihpe13030048

**Published:** 2023-03-15

**Authors:** Ibrahim A. Elshaer, Alaa M. S. Azazz

**Affiliations:** 1 Management Department, College of Business Administration, King Faisal University, Al-Ahsaa 31982, Saudi Arabia; 2 Hotel Studies Department, Faculty of Tourism and Hotels, Suez Canal University, Ismailia 41522, Egypt; 3 Social Studies Department, College of Arts, King Faisal University, Al-Ahsaa 31982, Saudi Arabia; 4 Tourism Studies Department, Faculty of Tourism and Hotels, Mansoura University, Mansoura 35516, Egypt

**Keywords:** job insecurity, family financial pressure, depression, stress, anxiety, mental health, tourism employees, PLS-SEM

## Abstract

Many people are experiencing a lack of confidence in the security of their employment due to the COVID-19 pandemic, particularly employees in the tourism sector, which has caused adverse effects on their mental health. These adverse effects involve the management of stress, anxiety and depression, that may arise from the demands of the industry. However, few studies have explored how insecurity in the workplace and financial pressure from families affects mental health and can intervene in these relationships. In this study, the aim was to investigate job insecurity as an antecedent of employees’ mental health and family financial pressures as a moderator using a sample of 475 hotel and travel industry employees. The theoretical background of the study was built upon the theories of resource conservation and effort–reward imbalance. The participants completed an online survey that measured job insecurity, family financial pressure, depression, anxiety, and stress. The collected data were subjected to PLS-SEM data analysis. The findings of this study reveal that job insecurity had a significant influence on depression, anxiety, and stress among tourism employees, and family financial pressure worsened the negative consequences of job insecurity on mental health. This research highlights the significance of addressing the mental health of employees in the tourism sector after the COVID-19 pandemic, as well as the crucial role played by family financial pressures. The findings of this study highlight the importance of addressing job insecurity in the tourism industry and its impact on employees’ mental health. This could involve implementing policies and practices that enhance job security, such as providing more stable work schedules, better benefits packages, and greater opportunities for professional development. The results also underscore the need to take into account the role of family financial pressure in moderating the impact of job insecurity on mental health. Practitioners and policymakers in the tourism industry should consider ways to alleviate financial pressure on employees and their families, such as offering assistance programs, flexible work arrangements, and supportive company policies.

## 1. Introduction

Health crises (i.e., COVID-19) can have a substantial influence on the tourism industry and the employees who work within it [1,2,3]. The tourism industry is specifically vulnerable to interruptions caused by health crises because it relies heavily on the movement of people and the availability of travel destinations [1,3,4]. Health crises can also lead to changes in consumer behavior that can negatively affect tourism employees [5]. For example, during a health crisis, people may avoid travelling or choose alternative destinations, which can lead to a decrease in demand for tourism services [6]. This can result in several negative impacts on tourism employees, including job loss, decreased work hours, reduced pay, and poor mental health [1,3]. The consequences of health crises on tourism employees can also vary according to their demographic characteristics and job-related variables. For example, research has shown that tourism employees who were older, female, had family, lesser degrees of education and less income were more affected by health crises [6,7]. Additionally, tourism employees who have more precarious working conditions (e.g., low pay, low career control) may be more vulnerable to the negative impacts of a health crisis (i.e., COVID-19 pandemic) [8], and an increased fear of job insecurity.

Job insecurity describes the uncertainty and anxiety that employees experience regarding the stability and safety of their employment [9]. This can include concerns about layoffs, downsizing, or job loss [10]. Job insecurity causes negative consequences for both physical and mental health [11]. Furthermore, individuals who have suffered from job insecurity may experience symptoms of depression and anxiety, as well as decreased job satisfaction and motivation [12]. Given the increased job insecurity that tourism workers have suffered from the COVID-19 pandemic, it is likely that they are at an intensified risk for poor mental health consequences such as depression, anxiety, and stress [13]. However, tourism employees may also experience increased financial pressure from their family as a consequence of the pandemic [14], which could further worsen the adverse influence of job insecurity on workers’ mental health consequences.

Family financial pressure refers to the perceived financial difficulties that individuals experience as a result of their family responsibilities (e.g., supporting children, elderly parents) [15]. Research has revealed that family financial pressure is linked with increased psychological distress [16,17] and that it can moderate the link from job insecurity to mental health results [5]. The link from job insecurity to mental health consequences has been well established in the literature [5,9,18,19,20,21,22,23]. However, little research has explored the moderating role of family financial pressures. The main objective of this paper is to investigate the moderating role of family financial pressures on the relationship between job insecurity and depression, anxiety, and stress among tourism employees. It is important for policymakers and employers to consider these interrelationships and develop interventions and policies that can help reduce the adverse effects of health crises on the tourism business and its employees. This can include measures such as financial support for affected employees, job retraining programs and mental health support services. Additionally, it is crucial to observe the tourism industry’s long-term sustainability and develop strategies to help it better withstand future health crises.

## 2. Theoretical Framework and Hypotheses Formulation

### 2.1. Theoretical Framework

The worldwide economy has been notably affected by the COVID-19 pandemic, leading to increased job insecurity among many workers, including those in the tourism industry [1]. Tourism employees, such as hotel and travel agents, have been specifically impacted by the pandemic. Travel restrictions and social distancing procedures have significantly decreased tourism and hospitality activity [8,24,25]. As a result, many tourism employees have experienced job loss, reduced work hours, and reduced pay [1,3,7]. Research has shown that insecurity in the workplace adversely influences mental health, including depression, anxiety, and stress [9,18]. Given the increased job insecurity that tourism employees have experienced from the COVID-19 pandemic, it is likely that they are at an increased risk for poor mental health outcomes.

The theoretical background of the current research paper may be built upon the conservation of resources theory (COR) [26,27], which posits that people have a limited amount of resources (e.g., time, money, social support) that they must allocate across various domains (e.g., work, family, health). According to this theory, job insecurity can be seen as a threat to an individual’s resources, as it may lead to the loss of financial resources and social support from colleagues. As a result, individuals may experience increased stress and decreased wellbeing. Another theory that has been used to explain the link between job insecurity and mental health is the effort–reward imbalance theory [28,29]. This theory proposes that when individuals perceive a mismatch between the effort they put into their work and the rewards they receive, they may experience stress and decreased wellbeing. By threatening an individual’s job and financial security, job insecurity may be perceived as an effort–reward imbalance, leading to negative mental health outcomes [30].

### 2.2. Hypotheses Formulation

#### 2.2.1. Job Insecurity as an Antecedent Mental Health

The examination of employees’ job uncertainty in the academic field started with a groundbreaking article by Greenhalgh and Rosenblatt [11]. They described the idea of job insecurity as the feeling of being unable to keep a job position secure when it is under threat. This perception can lead to negative reactions and outcomes for both individuals and businesses, such as decreased commitment, decreased satisfaction, and negative impacts on physical and mental health. Additionally, there is a positive connection between employees’ perceptions of job insecurity and mental health levels. Job insecurity is a recognized source of stress and can result in chronic stress reactions [10,23,31]. Previous research (prior to the COVID-19 outbreak), had already highlighted the concern of job insecurity for employees caused by economic instability and global changes [2,18]. This is principally evident in the tourism and hospitality sector where temporary work and unpredictability are prevalent [32,33]. The COVID-19 pandemic has worsened insecurity perception, disrupting employees’ future plans and careers [32,33] and negatively affecting their psychological wellbeing, especially in the tourism industry in Egypt [34]. Studies have shown that employees in Canada [35] and India [36] also experienced job insecurity and stress during the pandemic.

Studies have demonstrated that job insecurity might cause unfavorable consequences for a person’s physical and mental health. Meta analysis of the consequences of job insecurity, for instance, found that job insecurity raises the risk of cardiovascular disease and depression [30,37,38]. Additionally, job insecurity has been connected to a number of negative psychological results, such as increased stress, anxiety, and burnout. Employee stress arises frequently due to job uncertainty. Stress is a natural reaction to perceived threats and can be advantageous in moderation, however, ongoing stress can harm both physical and mental wellbeing [30,39,40]. According to Selye [41], stress can be both beneficial, assisting with adjustment to situations, changes, and events, and detrimental, reducing productivity and impacting health negatively, even leading to collapse under severe pressure. Theorell et al. [42] found that psychological and social workplace stress, as well as depression, are substantial risk factors for mental and physical health issues. In accordance with COR theory, job insecurity causes stress as it endangers an employee’s resources, such as steady employment, financial stability, success, and self-worth [26]. Research has demonstrated that job insecurity can be a source of chronic stress and serious long-term health issues [9,43]. The hospitality industry has a high-stress level due to the focus on high guest satisfaction and active and flexible work [44,45,46]. Hospitality employees experience significant stress due to the uncertainty caused by job insecurity [47,48,49]. Work surroundings with job insecurity, such as during the COVID-19 crisis, can also lead to stress [35]. COVID-19 has caused hotel employees to experience high stress levels due to unemployment [47].

Employee anxiety is also a common symptom of job insecurity, which includes fear of losing their job, fear of being unable to find new employment, and fear of not being able to support themselves or their families. According to [8,22,33,48,49], job insecurity can lead to increased levels of anxiety, which can negatively impact employee wellbeing and job performance. Therefore, it has been proposed that, when the perception of job insecurity increases, the negative consequences of mental health also increase. Hence, we can suggest the following hypotheses:

**H1.**  *Job insecurity can positively increase depression among tourism employees.*

**H2.**  *Job insecurity can positively increase anxiety among tourism employees.*

**H3.**  *Job insecurity can positively increase stress among tourism employees.*

#### 2.2.2. The Moderating Role of Family Financial Pressure

Both employees and their loved ones experience financial challenges due to the significant amount of job uncertainty [50]. Though significant improvement has been created in recent years in the understanding of the effects of job insecurity on wellbeing, stress, and health [10,19], it is still challenging to determine causality. The strain of financial needs on a family, such as paying for necessities, education, bills, and healthcare, can worsen the reactions of job insecurity and increase financial difficulties [38,51]. Several studies have looked into the connection between job insecurity and financial wellbeing and the stress levels of employees, with some finding significant effects [51] and others finding no significant impact [38].

Poor financial circumstances can increase stress and can negatively impact health, family, and employment [52]. Studies have shown that low income and financial insecurity can cause stress, low self-esteem, and psychological problems [53,54]. The COR framework suggests that a person’s financial situation, including their ownership of resources, can impact their level of stress if resources are lacking [27]. The study by Arampatzi et al. [53] argues that an individual’s financial situation plays a key role in determining the psychological impact of a crisis on employees in Europe. During the COVID-19 pandemic, it was found that individuals with greater financial concerns have higher feelings of stress and anxiety, as reported by Wilson et al. [54]. Furthermore, the influence of job insecurity on anxiety is further intensified by financial concerns, as noted by Shoss [55]. Additionally, economic vulnerability, which is caused by financial issues, leads to an increase in job insecurity and stress.

**Figure 1:**
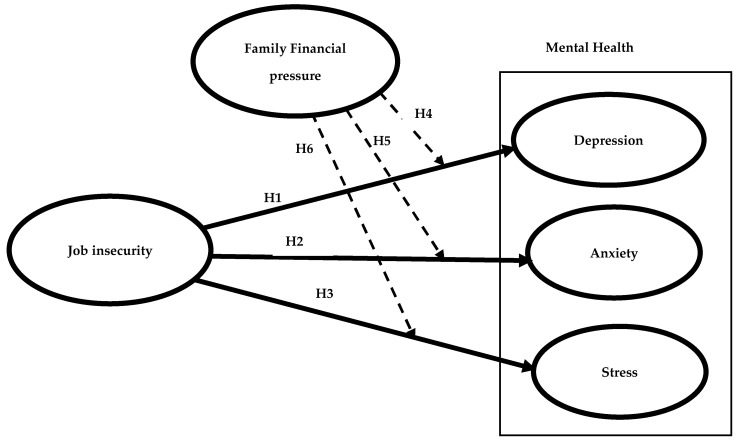
Research framework (dashed lines are moderating effects).

Research has shown that a person’s financial wellbeing can greatly impact their health. When someone experiences low financial wellbeing, such as from job insecurity, they may cut back on necessary expenses for health care and lose access to social insurance [56]. Additionally, financial stress can negatively affect a person’s physical and mental health, causing issues such as depression, stress and anxiety [57,58]. Accordingly, in our study, we propose that the negative impact of job insecurity on mental health outcomes will be stronger among those with higher levels of family financial pressure as below (See Figure 1):

**H4.**  *Family financial pressure moderates the link from job insecurity to depression among tourism employees.*

**H5.**  *Family financial pressure moderates the link from job insecurity to anxiety among tourism employees.*

**H6.**  *Family financial pressure moderates the link from job insecurity to stress among tourism employees.*

## 3. Methods

### 3.1. Data and Sample Procedure

A survey was conducted in the last two months of 2022 among 550 full-time employees of five-star hotels and category-A travel agents in Cairo and Sharm El Sheikh, both in Egypt. Tourism employees are an important population to study in this context as they are often exposed to job insecurity due to seasonality and economic fluctuations in the industry [34]. Furthermore, tourism employees may also experience additional financial pressure as they may be the primary breadwinners for their families and may be responsible for supporting multiple generations [1]. The study team created a survey using Google Forms and shared it through their personal networks on social media, resulting in a 95% response rate [59]. Of the 500 survey forms that were distributed, only 480 were completed and returned. Five of these were discarded as they were incomplete, leaving 475 questionnaires that could be used for analysis. Four hundred seventy-five participants as a sample size is adequate for PLS-SEM tests as it satisfies various sample size requirements for this type of study. It adheres to Nunnally’s [60] suggestion of having 10 participants per variable, as our scale has 28 indicators, surpassing the minimum requirement of 150. Additionally, our sample of 475 meets the requirement suggested by [61] to have at least 100 to 150 respondents for reliable estimation solutions and exceeds the recommended sample size suggested by [62] for study populations over 1,000,000, which is 384. Therefore, 475 as a sample size is sufficient for further analysis. A *t*-test was performed to decide if there were any major differences in the average responses between participants who completed the survey early and those who completed it later. The results showed no significant variations, indicating the possibility of non-response bias according to [63].

### 3.2. Study Scales

The scales utilized in the study were selected due to their recognized psychometric characteristics and were modified from prior research. The survey utilized a five-point Likert scale to assess the study’s measures. The DASS-21 scale, which measures depression, anxiety, and stress through 21 items and is practical for both non-clinical and clinical setting, was used to evaluate the mental health of employees. The DASS-21 is frequently utilized to detect negative emotions in individuals [64]. The DASS-21 has seven sub-components for each dimension. An illustration of depression sub-items includes statements such as “I couldn’t feel happy at all” and “I believed life had no purpose” (with a reliability Cronbach’s alpha *a* score of 0.927). Sample indicators of anxiety are “I felt near panic” and “I felt frightened for no apparent reason” (a reliability score of 0. 0.882). Examples of stress sub-items are “I struggled to calm down” and “I had trouble unwinding” (with a reliability score of 0.923). Job insecurity was quantified using six variables, three of which assessed its quantitative aspects and the other three evaluated its qualitative aspects. The questionnaire items were first proposed by [19] and later adopted by [14]. An example item for job insecurity is “I worry about losing my job” (with a reliability score of 0.951). Furthermore, the concept of family financial stress was measured using three self-reported items based on [65] and utilized by [66] in the hotel industry. An example item is, “My family struggles to pay bills,” and “My family has limited funds at the end of each month” (with a reliability *a* value of 0.907).

Participants evaluated their viewpoints concerning the listed questions on a five-point scale, with 1 being “strongly disagree” and 5 indicating “strong agreement”. The questionnaire was tested by the researchers with a sample group consisting of ten scholars and 17 workers to guarantee its accuracy and clarity. The questionnaire remained unchanged after the pilot test. Respondents were told that the data collected were kept confidential and anonymous. The questionnaire’s reliance on self-reported data raised the likelihood of common method variance (CMV) as an issue [67]. To take action on this matter, a Harman’s single-factor test was executed. The factors were set to the value of one in the exploratory factor analysis (EFA) method using the SPSS program with no rotation and just one factor emerged, explaining 39% (<0.5) of the variance. This suggests that CMV is not a problem [68].

## 4. Data Analysis Approach

The study used PLS-SEM to test the hypothesis that family financial pressure mediates the relationship between job insecurity and mental health for tourism employees. PLS-SEM enables the analysis of a greater number of variables per dimension compared with other statistical methods such as CB-SEM. The study utilized Leguina’s [69] two-part method, first evaluating the measurement model’s reliability and validity and then examining the structural model to support or reject the hypotheses. The outer model in PLS-SEM was evaluated using various suggested cut-off values, such as a standardized factor loading (SFL) of over 0.6, composite reliability (CR) of over 0.7, average variance extracted (AVE) of over 0.5, a normed fit index greater than 0.9, a standardized root mean square residual less than 0.08, R^2^ greater than 0.1 and Stone–Geisser Q^2^ higher than 0.0, as per Hair et al. [70].

## 5. Results

### 5.1. Descriptive Statistics

The results show that 72% of the participants were male and 28% were female. The age distribution was 28% under 20, 42% between 26–35, 15% between 36–45, and 15% 46 or older. The findings suggest that the majority (70%) of workers in Egypt’s tourism industry are young and capable of performing physically challenging tasks. Education-wise, 30% of participants had only completed secondary school or less, 60% had a bachelor’s degree, and only 10% held a postgraduate degree. Of the participants, 59% (280) came from five-star hotels and 41% (195) were from the travel agent category A. The participants were all non-managerial employees with 70% in front-line roles and 30% in back-line roles. In terms of experience, 25% had less than 1 year, 35% had 2–4 years, 30% had 5–7 years, and 10% had 8 or more years.

### 5.2. Outer Model Findings

Through various tests, the validity and reliability of the measurement model were assessed in the study, as depicted in Table 1. These tests included composite reliability (CR), Cronbach’s Alpha, and construct validity checks through discriminant and convergent validity. Table 1 reveals the psychometric properties of the employed measures for the job insecurity, family financial pressure, stress, anxiety, and depression latent dimensions (CR, Cronbach’s alpha, and AVE) all surpass the recommended threshold. The values are job insecurity (a = 0.951, C.R 0.953, AVE = 0.802), family financial pressure (a = 0.907, C.R. = 0.920, AVE = 0.843), stress (a = 0.923, C.R. = 0.928, AVE = 0.724), anxiety (a = 0.882, C.R. = 0.903, AVE = 0.637), and depression (a = 0.927, C.R. = 0.935, AVE = 0.733). All exceeded the suggested standard as suggested by hair (2014). The findings suggest that the study has robust internal consistency and convergent validity for the constructs.

Additionally, the factor loadings were all above 0.65, which supports the scale’s reliability as per Hair et al. [70]. The discriminant validity was also assessed using three methods from Leguina [69]: cross-loading, the Fornell–Larcker criterion, and the heterotrait–monotrait ratio. Table 2 shows that each latent variable’s items had higher loadings than cross-loadings with other items, demonstrating the distinctness of each latent variable.

The study analyzed various aspects of validity, including cross-loading and the Fornell–Larcker criterion. It also used the heterotrait–monotrait ratio test to assess construct discriminant validity. The results, presented in Table 3, suggest that the reliability and validity of the scales are adequate, as the squared AVE scores for each latent variable are higher than the inter-variable correlations and the heterotrait–monotrait ratio is below 0.90, as suggested by Leguina [69]. This allows for confident analysis of the inner model and hypothesis testing.

### 5.3. Inner Model Hypotheses Testing

The structural model was tested for its ability to predict the impact of exogenous latent variables on the latent dependent variables of depression, anxiety, and stress. Goodness of fit was measured using various metrics, and a minimum R2 score of 0.10 was considered acceptable according to Hair et al. [71]. The results show that the model had good predictive power with R2 values of 0.225, 0.129, and 0.277 for depression, anxiety, and stress, respectively. The Stone–Geisser Q^2^ criterion also supported the model’s accuracy as per Henseler et al. [72] with values of 0.188 for depression, 0.248 for stress, and 0.098 for anxiety.

**Figure 2:**
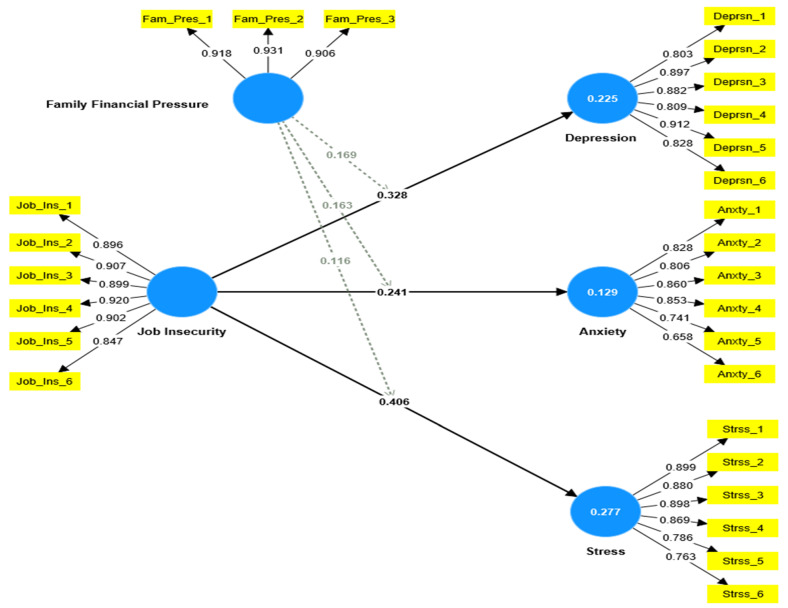
The PLS-SEM tested model.

The last phase of the analysis involved using a bootstrapping method with 5000 repetitions to determine the path coefficients and t-value significance for direct and moderating relationships, as displayed in Table 4 and Figure 2. The study put forth and tested three direct and three moderating hypotheses, which are depicted in Figure 2 and Table 4. The results show that employees’ perception of job insecurity had a positive and significant direct impact on all three negative mental health outcomes (depression β = 0.328, *t* = 6.123, *p* < 0.001), (anxiety β = 0.240, *t* = 4.476, *p* < 0.001), and (stress β = 0.406, *t* = 9.190, *p* < 0.001), thereby supporting hypotheses H1, H2, H3, and H4.

**Figure 3:**
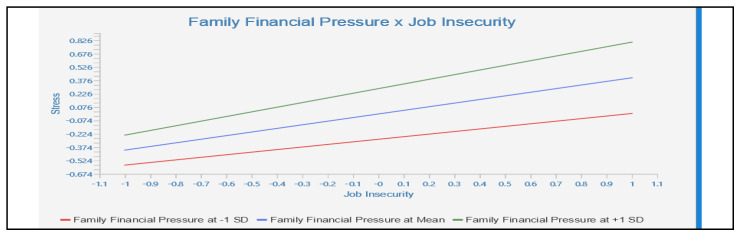

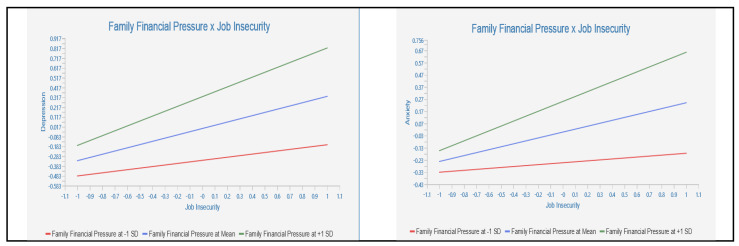
Simple slope results.

Figure 3 demonstrates the outcomes of the moderation assessment, which confirm that family financial pressures moderate the relationship under examination. The analysis performed using Smart-PLS demonstrated that family financial pressures significantly amplify the effect of job insecurity on employee experiences of depression (β = 0.169, *t* = 3.212, *p* < 0.001). This suggests that as family financial pressure increases, the effect of job insecurity on employee depression also increases, thereby supporting H4. Likewise, the results indicate that increased family financial pressure intensifies the influence of job insecurity on employee perceptions of anxiety (β = 0.163, *t* = 3.518, *p* < 0.001) and stress (β = 0.116, *t* = 2.238, *p* < 0.05). This enables us to affirm H5 and H6.

## 6. Discussion

Tourism is a fast-growing industry, contributing significantly to the global economy. The COVID-19 pandemic has caused a significant decline in the tourism industry, leading to widespread job insecurity among its employees. Job insecurity is frequently linked to negative outcomes for employees’ mental health. The impact of job insecurity on employee mental health has been well documented in the literature, and the COVID-19 pandemic has only amplified this issue. The uncertainty and fear of job loss have significant consequences for employees, including increased levels of stress, anxiety, and depression [11]. In the post-COVID era, job insecurity has caused significant worry for employees worldwide, as the pandemic has resulted in widespread layoffs, furloughs, and wage cuts [73]. Previous research has tested the interrelationship between job insecurity and various negative effects on employees’ mental health [21,74,75,76,77], but the connection and interaction between the two is unclear. Our research seeks to fill a gap in the existing literature by examining the effects of family financial pressures on the correlation between job insecurity and mental health outcomes such as depression, anxiety, and stress, which has not yet been explored.

Data were collected from 475 non-managerial hotel and travel agent employees. The collected data were analyzed by PLS-SEM, GmbH, Germany. The findings of our study revealed that job insecurity was positively related to poor mental health outcomes among tourism employees. The results suggest that job insecurity is a significant concern for tourism employees post COVID-19 pandemic and may contribute to increased levels of stress. This result is consistent with [78], who found that job insecurity among tourism employees during the COVID-19 pandemic was significantly related to higher levels of stress, depression, and anxiety. The authors argue that the lack of stability and uncertainty regarding job status and future employment opportunities have contributed to increased psychological distress among these workers. The findings of our study confirm the significant positive effects of job insecurity as well on employee’s perception of depression and anxiety. These results are in line with [8,13,33] who found that job insecurity is a significant predictor of depression and anxiety, and that these effects are more pronounced among workers in the service sector, including tourism. The study highlights the need for interventions to address job insecurity and its associated negative effects on the mental health of tourism employees. Furthermore, employees who are under higher levels of financial pressure are more likely to experience negative mental health outcomes as a result of job insecurity (Kim and Moon, 2020). In other words, the path from job insecurity to mental health may be stronger for employees who are facing financial difficulties, as they are more likely to feel a greater sense of helplessness and hopelessness [39,79]. Given the moderating impacts of family financial pressures, organizations should consider financial support for employees, in addition to measures aimed at reducing job insecurity.

Several theoretical and practical consequences arose from our study. From a theoretical view, the moderating impacts of family financial pressures highlights the interplay between various factors that can impact employee mental health. Our research suggests that employees under higher levels of financial pressure are more likely to experience negative mental health outcomes as a result of job insecurity. This finding contributes to the ever-expanding literature that proposes that the relationship between job insecurity and mental health is not straightforward and is influenced by a complex interplay of factors, including financial strain, social support, and coping resources [11]. Furthermore, the moderating influence of family financial pressures highlights the importance of considering employees’ broader life context when examining the impact of job insecurity on mental health [14]. This finding underscores the need for a holistic approach to employee wellbeing, one that considers not only work-related stressors but also broader life stressors that can impact mental health.

Regarding practical implications, organizations can support their employees by addressing job insecurity and offering resources and support for those facing financial difficulties. This can include providing flexible work arrangements, such as reduced hours or telecommuting, and access to financial planning and counselling services. Organizations can also prioritize employee wellbeing by regularly checking in, offering mental health support, and creating a supportive and inclusive workplace culture [80]. There are several other strategies that organizations can implement to provide support to employees during this time of increased job insecurity. Some of these strategies include providing clear and transparent communication. Regular and open two-way communication with employees can help reduce uncertainty and anxiety. Organizations can provide regular updates on the state of the business, including any changes to staffing or compensation, to help employees feel informed and supported. Providing opportunities for training and development can help employees to feel valued and secure in their roles, even during times of uncertainty. This can include offering training in new skills or certifications that can help employees to remain competitive in the job market, while encouraging employees to connect and support one another can help to foster a sense of community and reduce feelings of isolation. This can include hosting virtual events or encouraging employees to form online support groups. By implementing these strategies, organizations can support their employees during this difficult time and help to alleviate the adverse influence of job insecurity on mental health.

Our study, like others, has limitations in its generalizability to other cultures, causality and temporal sequence, and potential bias from self-reported data. To enhance the generalizability of the study results, comparable research can be carried out in other countries or areas with distinct cultural contexts. Further research using multiple methods (i.e., using mixed-methods approaches or conducting independent replications of their findings) is needed to increase validity. The relationship between job insecurity, family financial pressure and employee mental health in the tourism industry is a complex and multi-layered area that requires more in-depth study. Future studies should use different research designs, consider cultural contexts, and examine other factors affecting mental health. Future research should also assess the role of coping resources, such as social support, financial resources, and coping strategies, in mediating the relationship between job insecurity and mental health. Future research should use longitudinal designs to examine the impact of job insecurity on mental health over time and to establish causality. Additionally, the model can be extended to examine the impact of job insecurity on turnover intention [81] through the mediating role of mental health. Finally, future research should examine the impact of job insecurity on mental health in diverse populations, including individuals from different age groups, genders, races/ethnicities, and socioeconomic statuses.

## 7. Conclusions

Job insecurity is a prevalent issue that significantly impacts hotel employees’ mental health and wellbeing. Despite the vast body of research on workplace insecurity, there has been limited investigation into its impact on mental health and how family financial pressures could potentially moderate these effects. Four hundred seventy-five five-star hotel employees participated in this study, the collected data were evaluated with regard to discriminant and convergent validity and the proposed hypotheses were tested using the SmartPLS 4 program, GmbH, Germany. The study found that job insecurity was associated with negative mental health outcomes, including higher levels of psychological distress, depression, and anxiety. Furthermore, family financial pressures were found to moderate the relationship between job insecurity and mental health outcomes, suggesting that financial support may help alleviate the negative effects of job insecurity on employees. These findings have significant implications for hotel managers and policymakers. They should prioritize implementing job security measures, such as offering stable work contracts and providing clear job expectations. Moreover, they should also consider the financial strains that employees may face, especially when job security is low, and offer financial support or counseling services to alleviate the impact on mental health. This study provides a foundation for further research and underscores the need for organizational interventions to address job insecurity and promote a healthy and productive workforce.

## Figures and Tables

**Table 1:** Psycometric properties of the study measures.

Dimension/Items	Loadings	α	C_R.	AVE
Cut-off values	>0.7	>0.7	>0.7	>0.5
Jon insecurity		0.951	0.953	0.802
Job_Ins_1	0.896			
Job_Ins_2	0.907			
Job_Ins_3	0.899			
Job_Ins_4	0.920			
Job_Ins_5	0.902			
Job_Ins_6	0.847			
Family financial pressure		0.907	0.920	0.843
Fam_Pres_1	0.918			
Fam_Pres_2	0.931			
Fam_Pres_3	0.906			
Depression		0.927	0.935	0.733
Deprsn_1	0.803			
Deprsn_2	0.897			
Deprsn_3	0.882			
Deprsn_4	0.809			
Deprsn_5	0.912			
Deprsn_6	0.828			
Anxiety		0.882	0.903	0.631
Anxty_1	0.828			
Anxty_2	0.806			
Anxty_3	0.860			
Anxty_4	0.853			
Anxty_5	0.741			
Anxty_6	0.658			
Stress		0.923	0.928	0.724
Strss_1	0.899			
Strss_2	0.880			
Strss_3	0.898			
Strss_4	0.869			
Strss_5	0.786			
Strss_6	0.763			

**Table 2:** Cross-loadings scores.

Factors/Items	Anxiety	Depression	Family Financial Pressure	Job Insecurity	Stress
Anxty_1	0.828	0.660	0.275	0.240	0.491
Anxty_2	0.806	0.467	0.132	0.172	0.287
Anxty_3	0.860	0.548	0.164	0.200	0.266
Anxty_4	0.853	0.529	0.197	0.218	0.349
Anxty_5	0.741	0.417	0.132	0.136	0.283
Anxty_6	0.658	0.404	0.163	0.201	0.367
Deprsn_1	0.615	0.803	0.284	0.372	0.537
Deprsn_2	0.579	0.897	0.328	0.339	0.619
Deprsn_3	0.528	0.882	0.283	0.324	0.598
Deprsn_4	0.579	0.809	0.276	0.225	0.517
Deprsn_5	0.528	0.912	0.335	0.361	0.640
Deprsn_6	0.525	0.828	0.272	0.263	0.529
Fam_Pres_1	0.232	0.325	0.918	0.491	0.394
Fam_Pres_2	0.234	0.369	0.931	0.466	0.360
Fam_Pres_3	0.173	0.252	0.906	0.486	0.329
Job_Ins_1	0.203	0.322	0.414	0.896	0.414
Job_Ins_2	0.209	0.325	0.427	0.907	0.434
Job_Ins_3	0.245	0.325	0.429	0.899	0.395
Job_Ins_4	0.266	0.370	0.475	0.920	0.458
Job_Ins_5	0.211	0.275	0.478	0.902	0.390
Job_Ins_6	0.216	0.374	0.579	0.847	0.421
Strss_1	0.373	0.534	0.348	0.431	0.899
Strss_2	0.292	0.496	0.327	0.419	0.880
Strss_3	0.382	0.540	0.363	0.448	0.898
Strss_4	0.325	0.510	0.316	0.394	0.869
Strss_5	0.446	0.667	0.324	0.338	0.786
Strss_6	0.473	0.729	0.340	0.352	0.763

**Table 3:** Claculations of discriminant validity.

	AVEs Matrix					HTMT Matrix				
	I	II	III	IV	V	I	II	III	IV	V
I. Anxiety	0.794									
II. Depression	0.652	0.856				0.705				
III. Family Financial Pressure	0.235	0.348	0.918			0.247	0.372			
IV. Job Insecurity	0.252	0.373	0.523	0.896		0.267	0.388	0.562		
V. Stress	0.445	0.673	0.395	0.469	0.851	0.481	0.734	0.430	0.497	

**Table 4:** Hypotheses evaluation.

Direct and Specific Indirect Effects	β	*t*-Value	*p*-Value	Results
**Direct effects**				
Job Insecurity -> Depression	0.328	6.123	0.000	H1 Supp.
Job Insecurity -> Anxiety	0.241	4.476	0.000	H2 Supp.
Job Insecurity -> Stress	0.406	9.190	0.000	H3 Supp.
**Moderating effects**				
Family Financial Pressure x Job Insecurity -> Depression	0.169	3.212	0.001	H4 Supp.
Family Financial Pressure x Job Insecurity -> Anxiety	0.163	3.518	0.000	H5 Supp.
Family Financial Pressure x Job Insecurity -> Stress	0.116	2.238	0.025	H6 Supp.

## Data Availability

Data are available upon request from researchers who meet the eligibility criteria. Kindly contact the first author privately through e-mail.
